# Estimation of regional-scale maize plant nitrogen content based on multi-source remote sensing data

**DOI:** 10.3389/fpls.2025.1669170

**Published:** 2025-09-26

**Authors:** Jixuan Yan, Yayu Wang, Zichen Guo, Wenning Wang, Yinshan Ma, Jie Li, Xiangdong Yao, Qiang Li, Kejing Cheng, Guang Li, Weiwei Ma

**Affiliations:** ^1^ State Key Laboratory of Aridland Crop Science, Gansu Agricultural University, Lanzhou, China; ^2^ College of Water Conservancy and Hydropower Engineering, Gansu Agricultural University, Lanzhou, China; ^3^ College of Agriculture and Ecological Engineering, Hexi University, Zhangye, China; ^4^ College of Forestry, Gansu Agricultural University, Lanzhou, China

**Keywords:** PNC, UAV, Sentinel-2A, regional scale, inversion

## Abstract

This study aims to systematically analyze the challenges of water scarcity and low nitrogen use efficiency in maize production in the arid Hexi Corridor. It provides a scientific basis for efficient water and fertilizer management. This study innovatively integrates multi-source data from satellite and Unmanned Aerial Vehicle (UAV) remote sensing. The datasets include Sentinel-2A imagery, UAV-based multispectral images, and ground-based observations. Based on these data, a comprehensive data fusion framework was established. Data were collected across four key growth stages of maize in 2024, with 66 sampling points established in the main experimental area and 48 sampling points in the auxiliary validation area for model training and validation. Pearson correlation analysis was employed to identify the optimal combination of vegetation indices (VIs). The inversion accuracy of various models at different growth stages was systematically analyzed. Notably, a novel region-scale maize Plant Nitrogen Content (PNC) inversion method based on band correction was proposed. This method not only achieves the harmonization of multi-source remote sensing data but also optimizes the PNC inversion at the regional scale, accounting for inter-sensor spectral response differences and leveraging multi-growth-stage data to enhance the model’s robustness and generalization capability. Furthermore, the applicability and reliability of this model for crop growth monitoring in arid regions were thoroughly evaluated. The results showed that: (1) The PNC prediction model based on Convolutional Neural Networks (CNN) demonstrated significant performance advantages. It achieved a coefficient of determination (R²) of 0.80. Compared with traditional machine learning models, such as Support Vector Machines (SVM) and Random Forest (RF), the prediction accuracy improved by more than 10%. (2) Band correction significantly enhanced the modeling performance of Sentinel-2A data in PNC retrieval. The R² of the prediction model increasing from 0.35-0.45 (uncorrected) to 0.70-0.80. This confirmed the positive impact of band correction on model accuracy. (3) The prediction accuracy in the auxiliary validation area was highly consistent with that in the main validation area, further confirming the stability and reliability of the proposed method under varying regional conditions. This study provides an effective approach for rapid and precise monitoring of maize nitrogen status in arid regions. It also offers scientific support for regional-scale crop nitrogen management and precision fertilization decisions. The findings have significant theoretical and practical implications.

## 1 Introduction

Nitrogen content is a key biochemical indicator for assessing crop growth, development, and yield potential (Plénet and Lemaire, 1999). Nitrogen deficiency significantly inhibits the biosynthesis of amino acids, proteins, and chlorophyll within plants, thereby reducing photosynthetic efficiency and affecting plant growth and yield formation (Ding et al., 2005). Maize is one of the most important food crops globally and serves as a key source of feed and bioenergy. Efficient nitrogen fertilizer management is crucial for ensuring food security and improving yield and quality (Baum et al., 2025). In arid and semi-arid regions, water scarcity and low nitrogen use efficiency severely constrain crop production, and these challenges are further exacerbated by climate change (Shayanmehr et al., 2022). Therefore, efficient and rapid monitoring of crop nitrogen content is of significant theoretical and practical importance for optimizing nitrogen fertilizer management strategies, predicting yield potential, and formulating scientific field water and fertilizer regulation plans (Xiao et al., 2025).

Traditional methods for determining crop nitrogen content mainly rely on destructive sampling and laboratory analysis, which are time-consuming, labor-intensive, and lack timeliness, making large-scale non-destructive diagnosis unfeasible and hindering the development of large-scale agriculture (Ali et al., 2017). Traditional nitrogen content monitoring primarily relies on destructive sampling and laboratory chemical analysis methods (Sáez-Plaza et al., 2013). These methods are not only time-consuming and inefficient in terms of sampling but also can only cover a limited number of discrete points, making it difficult to achieve spatial continuity at the field scale. As a result, they are inadequate for providing comprehensive and effective monitoring over large agricultural areas (Swathy et al., 2024). In contrast, satellite remote sensing technology offers wide-area coverage, enabling continuous observation at regional to global scales (Sishodia et al., 2020). However, due to limitations in revisit cycles and factors such as cloud cover, mixed pixel effects often occur in medium- to small-scale agricultural monitoring (Mehedi et al., 2024), resulting in decreased accuracy of the inversion of key agricultural parameters. This reduces its ability to meet the precise monitoring needs of crop growth at the field scale, thus limiting its application in agriculture. UAV remote sensing technology, with its advantages of low cost, high spectral resolution, and easy accessibility (Zheng et al., 2021), is capable of capturing high-precision crop canopy imagery over small areas, effectively addressing the limitations of satellite-based remote sensing platforms in agricultural monitoring (Phang et al., 2023). Therefore, by integrating satellite and UAV remote sensing technologies and establishing a multi-source collaborative observation system, the wide-area imaging capability of the satellite platform and the high spatial resolution (centimeter-level) spectral data from UAVs complement each other effectively, significantly optimizing the regional applicability of crop growth models (Alexopoulos et al., 2023). Moreover, this multi-source fusion strategy not only improves the spatiotemporal continuity and accuracy of monitoring, but also enhances the characterization of crop physiological and ecological processes under complex agricultural conditions (Dong et al., 2017). This approach provides key technical support for building an integrated “space-air-ground” agricultural monitoring system and offers new perspectives and methods for regional-scale precision crop management and sustainable development (Zhang et al., 2019).

The synergy between satellite and UAV observations demonstrates significant advantages in the quantitative retrieval of crop physiological and biochemical parameters. This technical framework integrates remote sensing data with varying spatial, temporal, and spectral resolutions, effectively overcoming the limitations of using a single data source, such as timeliness and long-term continuous monitoring (Platel et al., 2025). It significantly enhances the retrieval accuracy and robustness of key agricultural parameters. Qi et al. (2020) constructed a satellite-UAV-ground integrated monitoring system and combined an improved soil salinity sensitivity index with a Random Forest algorithm to achieve accurate soil salinity estimation for winter wheat in the Yellow River Delta. This study demonstrates that integrating multi-source collaborative observations with machine learning can significantly improve the retrieval accuracy of key crop parameters. It provides a methodological reference for the present study in applying satellite-UAV data fusion combined with machine learning for regional-scale maize PNC retrieval. Li et al. (2022) proposed a spatiotemporal monitoring framework that integrates high-resolution UAV imagery with Sentinel-2 satellite data. By assigning different weights to each data source, the framework improved the accuracy of winter wheat growth monitoring during key developmental stages and provided a new approach for multi-scale crop growth assessment. Their findings indicate that multi-platform coordination is an effective approach to improving the accuracy of regional agricultural monitoring. Qi et al. (2022) proposed an integrated approach combining satellite-ground spectral fusion and satellite-UAV data fusion. By optimizing the selection of key spectral bands, this method significantly improved the accuracy of soil salinization monitoring in coastal cotton fields. Their study demonstrates that multi-source data fusion and feature optimization play a significant role in improving the accuracy of regional crop physiological parameter monitoring, which closely aligns with the objective of our study to retrieve maize PNC through satellite-UAV synergy. Yuan et al. (2025) developed a monitoring framework for rice blast disease by integrating high-resolution UAV imagery with multispectral satellite data. This approach enabled early detection of the disease from field to regional scales with an accuracy of 89.2%, offering an effective solution to the trade-off between spatial–temporal resolution and coverage inherent in single-platform remote sensing systems. This study’s exploration of using multi-source remote sensing data fusion to overcome bottlenecks in agricultural monitoring is highly aligned, at the methodological level, with our core approach-integrating the broad coverage of satellites with the high-resolution observations of UAVs to achieve high-accuracy regional-scale maize nitrogen content retrieval. Although previous studies have demonstrated the clear advantages of integrating satellite and UAV observations, limitations remain in regional-scale applications, multi-growth-stage data integration, and the handling of spectral differences between sensors. This study aims to address these issues by developing a band-corrected, regional-scale maize PNC retrieval method that integrates multi-source and multi-temporal data to enhance model stability and generalizability.

This study was conducted in three representative plots located in Minle County, Zhangye City, Gansu Province. A multi-platform observational framework integrating field sampling, UAV, and satellite imagery was established to investigate the inversion of PNC in maize. The aim was to achieve high-precision PNC estimation through the integration of multi-source data. The specific goals were as follows: (1) Develop an improved satellite-UAV band correction method and incorporate a sample augmentation strategy to construct PNC retrieval models based on different remote sensing data sources. (2) Systematically evaluate the accuracy and applicability of the PNC retrieval models across different data sources, highlighting the strengths and limitations of each model. (3) Extract the maize planting areas in Minle County using remote sensing image classification techniques and apply the optimal PNC retrieval model to generate county-scale PNC distribution maps, providing scientific basis and technical support for regional nitrogen monitoring and management.

## 2 Data sources

### 2.1 Study area overview

This study was conducted at Huarui Farm, Minle County, Zhangye City, Gansu Province, China (100°36′-101°03′E, 38°22′-38°48′N). Minle County is located in the central part of the Hexi Corridor, on the northern foothills of the Qilian Mountains, and is characterized by a typical temperate continental arid climate (Jiang et al., 2022). According to meteorological data from the Minle County Weather Station over the past decade, the region has an average annual temperature of 5.2 °C and an average annual precipitation of 286.5 mm, with approximately 60% falling between July and September. The annual evaporation reaches 2036.8 mm, resulting in a dryness index (K) of 4.3, indicating a pronounced imbalance between water supply and heat demand (Gao and Zhang, 2016). The soil in the study area is primarily composed of grey-cinnamon and irrigated alluvial soils. The 0–20 cm topsoil layer contains 12.3 ± 2.1 g/kg of organic matter and has a pH of 8.2 ± 0.3, exhibiting the typical physicochemical characteristics of arid soils in northwestern China (Liu et al., 2014). This region is characterized by scarce precipitation and intense evaporation. The limited rainfall combined with high evaporative demand makes agricultural production highly dependent on irrigation. Meanwhile, the soil exhibits low fertility and nutrients are prone to leaching, posing considerable challenges for nitrogen management. As maize is a key crop for both grain production and seed cultivation in this area, its growth and development are highly sensitive to water and nitrogen supply. Therefore, conducting remote sensing-based nitrogen content monitoring and inversion research in this region is not only beneficial for improving nitrogen use efficiency but also holds significant importance for food security and the advancement of precision agriculture in arid zones.

**Figure 1:**
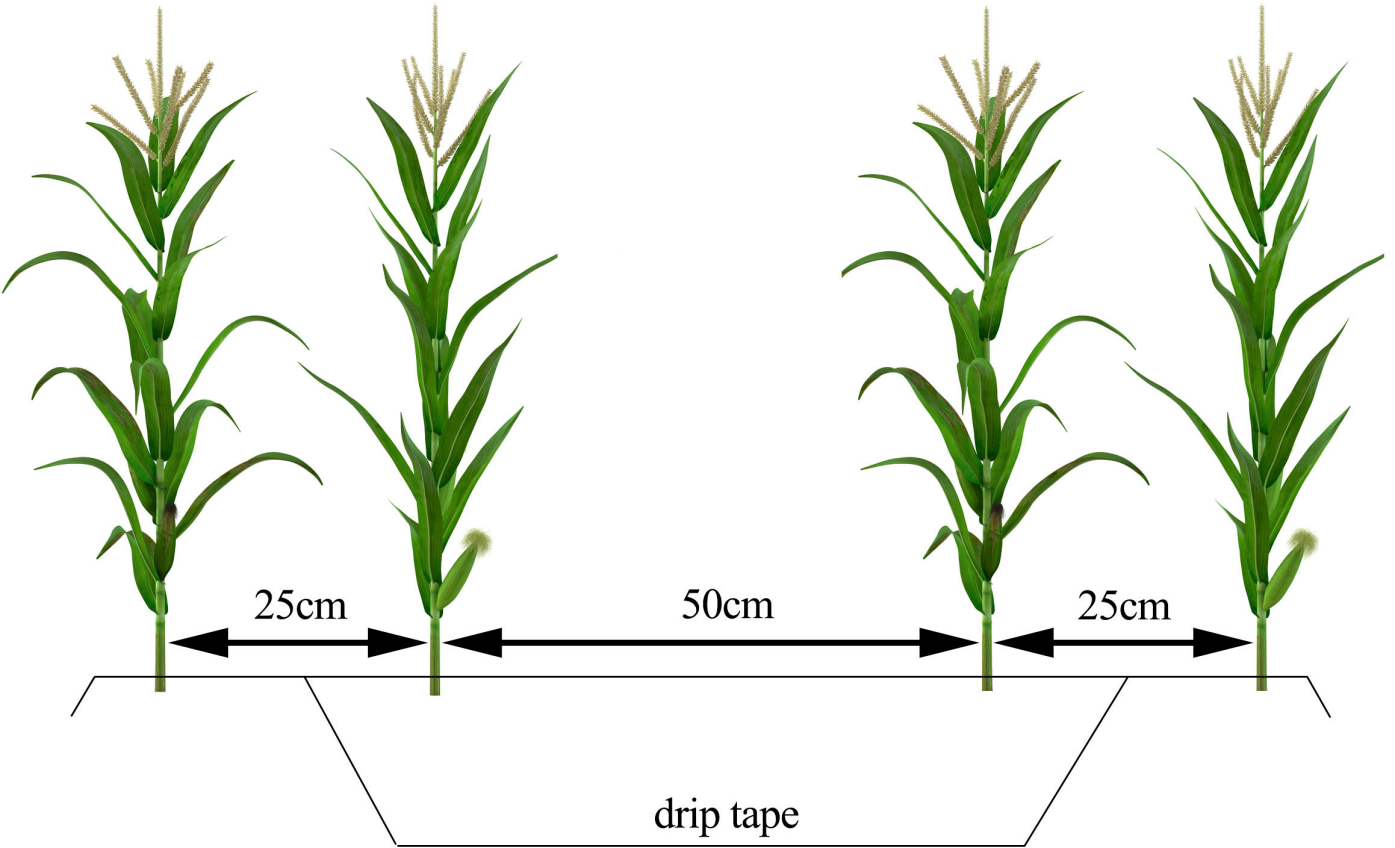
Maize cultivation patterns.

**Figure 2:**
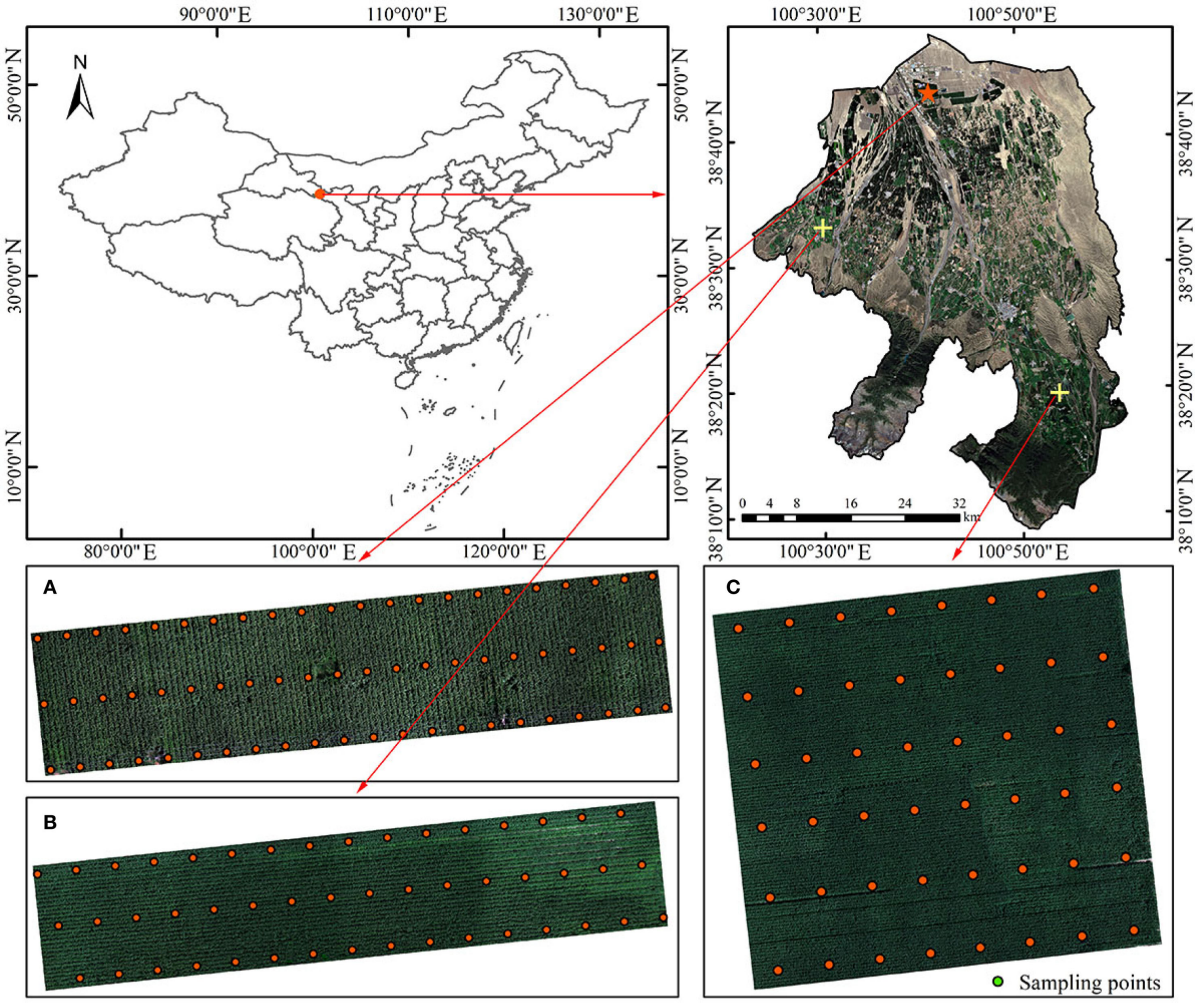
Overview of the study area. **(A)** Main experimental area. **(B, C)** Auxiliary experimental areas.

In this study, three representative maize cultivation plots in Minle County were selected as experimental areas, covering an elevation gradient ranging from 1768 to 2143 meters. All plots followed a typical local intensive management regime (**Figure 1**), including full-mulch double-ridge furrow sowing, a planting density of 6.75×10^4^ plants/ha, drip irrigation during the growing season, and nitrogen application at 280 kg/ha. **Figure 2A** shows the main experimental area, where 66 sampling points were established with an inter-point distance of approximately 5–11 m, and coordinates were precisely determined using GPS. **Figures 2B, C** depict the auxiliary validation areas, with 48 sampling points each, spaced approximately 9–12 m and 16–22 m apart, respectively, also located using GPS. The sampling points were selected to cover different soil types and growth conditions, ensuring that the samples adequately represent the characteristics of the region.

### 2.2 Data sources and preprocessing

#### 2.2.1 PNC of maize

To minimize edge effects and potential interference from adjacent fields, sampling points were positioned at least 0.5 m away from the edge of the plots. The maize PNC measured in this study refers to the nitrogen content of the whole aboveground plant, with the sampling and measurement standards as follows: In each experimental subplot, three centrally located maize plants with uniform growth and intact canopies were selected. After removing the roots, plants were separated into stem, leaf, and ear organs. Samples were first sterilized at 105°C for 30 minutes, then oven-dried at 85°C to a constant weight. The dried samples were ground, thoroughly homogenized, and digested using a mixed H_2_SO_4_-H_2_O_2_ solution. The nitrogen concentration of the maize plants was subsequently determined using the Kjeldahl method.

**Figure 3:**
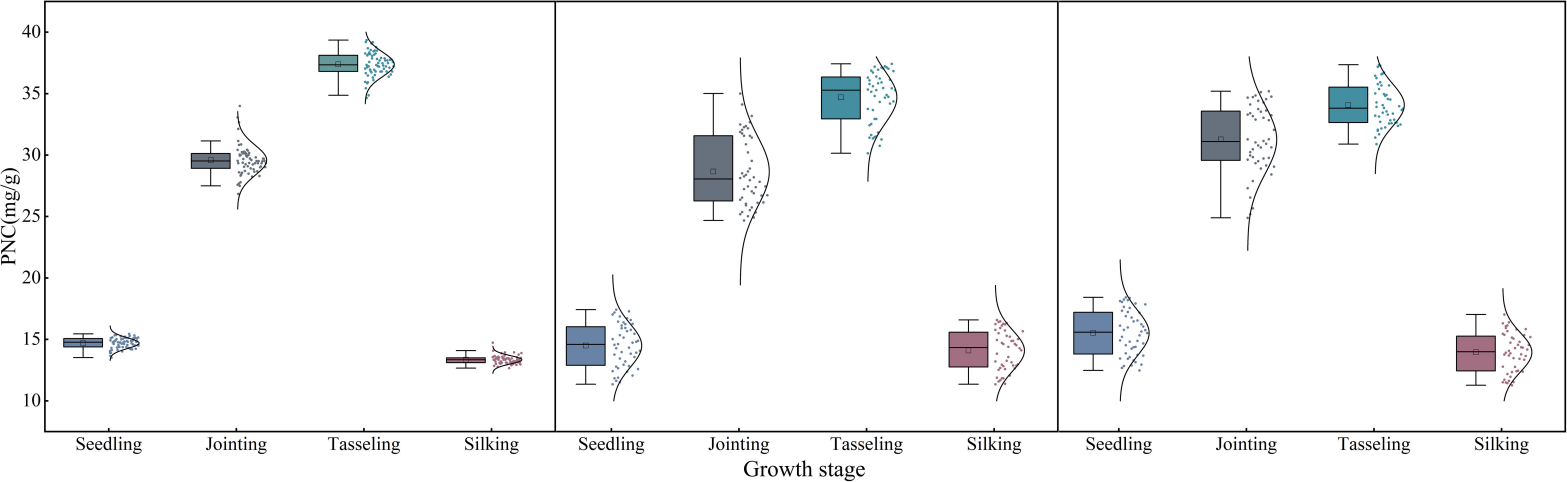
Statistical analysis of maize PNC.

**Figure 3** presents boxplots illustrating the distribution characteristics of maize PNC data across plots A, B, and C, clearly depicting the degree of dispersion and the presence of outliers in each plot. Outliers detected in the figure were processed using a robust normalization method. The criterion for identifying outliers was data points deviating from the median by ±1.5 times the interquartile range (IQR). By replacing the conventional mean and standard deviation with the median and IQR, this approach effectively enhances robustness against outliers, thereby improving the stability and reliability of data analysis (Rousseeuw and Croux, 1993). The processed dataset not only minimizes the influence of outliers on the overall distribution but also preserves the inherent biological variability of the samples (Filzmoser et al., 2008).

In this study, a systematic sampling approach was employed to divide the dataset. All sample data (n = 66/48) were first sorted in ascending order based on observed values. A fixed interval of k = 3 was then applied to perform equidistant sampling, where every third sample-starting from the first element-was selected into the validation set, resulting in 22/16 validation samples. The remaining 44/32 unselected samples were assigned to the modeling set. This method ensured full-range data coverage, thereby maintaining statistical consistency between the validation and modeling sets and effectively avoiding local clustering that may occur in random sampling.

#### 2.2.2 UAV data

**Figure 4:**
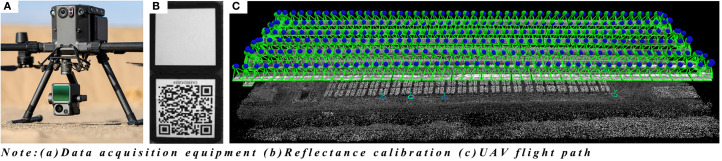
Data acquisition system. **(A)** Data acquisition equipment **(B)** Reflectance calibration **(C)** UAV flight path.

This study employed the DJI M300 RTK UAV platform equipped with the MS600 Pro multispectral sensor to conduct systematic multispectral remote sensing observations of maize during key growth stages, including the seedling stage (May 22), jointing stage (June 21), tasseling stage (July 26), and silking stage (August 10). To ensure data quality and consistency, all UAV aerial survey operations strictly adhered to predefined environmental and flight conditions. Missions were conducted on clear days without precipitation and with good visibility, within the local time window of 11:00-13:00 to minimize shadow effects caused by variations in solar zenith angle. Ground wind speed was strictly controlled below 3 m/s to guarantee geometric stability of the imagery. The flight was performed at a fixed altitude of 60 m, with forward and side overlap rates set to 80% and 70%, respectively. A total of six flight lines were planned at a speed of 1.5 m/s, resulting in imagery with a spatial resolution of 0.04 m. Furthermore, to maximize the synchronization between remote sensing data and ground truth, all plant sampling was completed within 30 minutes after the corresponding data acquisition. The MS600 Pro multispectral camera was equipped with six bands corresponding to blue, green, red, reg1, reg2, and near-infrared. The UAV flight missions lasted approximately 30 minutes. A standardized radiometric correction procedure was employed to ensure the consistency of multi-temporal remote sensing data. Specifically, ground correction panel imagery was synchronously acquired before each flight mission to facilitate reflectance correction (**Figure 4B**). The use of correction panels effectively mitigated radiometric distortions caused by variations in solar zenith angles and fluctuations in atmospheric conditions, compensating for radiometric differences across different observation periods and dates. By normalizing the target reflectance with respect to the correction panel reference values, the comparability of multi-temporal data and the accuracy of quantitative inversion were significantly improved, providing a reliable radiometric baseline for time-series analysis.

The Pix4D Mapper software was used to perform bundle block adjustment and generate Digital Orthophoto Maps from UAV images acquired at different growth stages (**Figure 4C**). The raw multispectral images were processed in ENVI 5.6 by performing band composition to generate standard false-color images. Supervised classification was conducted using the Maximum Likelihood Classification method, with representative soil and shadow samples selected as regions of interest to identify and remove non-vegetation background. Using the Raster Calculator and Extract by Mask tools in ArcMap 10.8, the images were precisely clipped according to the vector boundaries of the study area, resulting in clean maize canopy reflectance data. This workflow extracted six spectral bands of maize canopy reflectance for each growth stage, providing standardized input for subsequent vegetation index calculation and nitrogen content estimation.

#### 2.2.3 Sentinel-2A satellite data

Sentinel-2A, an important Earth observation satellite of the European Space Agency (ESA) under the Copernicus program, was launched on June 23, 2015, and, together with Sentinel-2B, forms a dual-satellite system (Drusch et al., 2012). Equipped with a Multispectral Sensor Imaging (MSI), Sentinel-2A is capable of capturing images across 13 spectral bands, spanning from visible to shortwave infrared wavelengths. Each single scan covers a width of 290 km, enabling the acquisition of large-scale surface images in a short time. The image data for the study area covers the key maize growth stages, and the satellite band parameters selected based on spectral matching are shown in **Table 1**.

**Table 1:** Spectral band information of UAV and Sentinel-2A imagery.

UAV				Sentinel-2A			
Band	Band center/nm	Band width/nm	Resolution/m	Band	Band center/nm	Band width/nm	Resolution/m
BLUE	450	35	0.04	B2	490	65	10
GREEN	555	25	0.04	B3	560	35	10
RED	660	20	0.04	B4	665	30	10
REG1	720	10	0.04	B5	705	15	20
REG2	750	15	0.04	B6	740	15	20
NIR	840	35	0.04	B8	842	115	10

In this study, surface reflectance products of Sentinel-2A Level-2A-preprocessed by radiometric correction and atmospheric correction-were acquired via the Google Earth Engine (GEE) cloud computing platform (https://code.earthengine.google.com) (Gorelick et al., 2017). Four Sentinel-2A scenes covering the entire Minle County were extracted through spatial domain filtering. All spectral bands were resampled to a unified spatial resolution of 10 meters to ensure consistency in spatial scale across multiple data sources.

**Figure 5:**
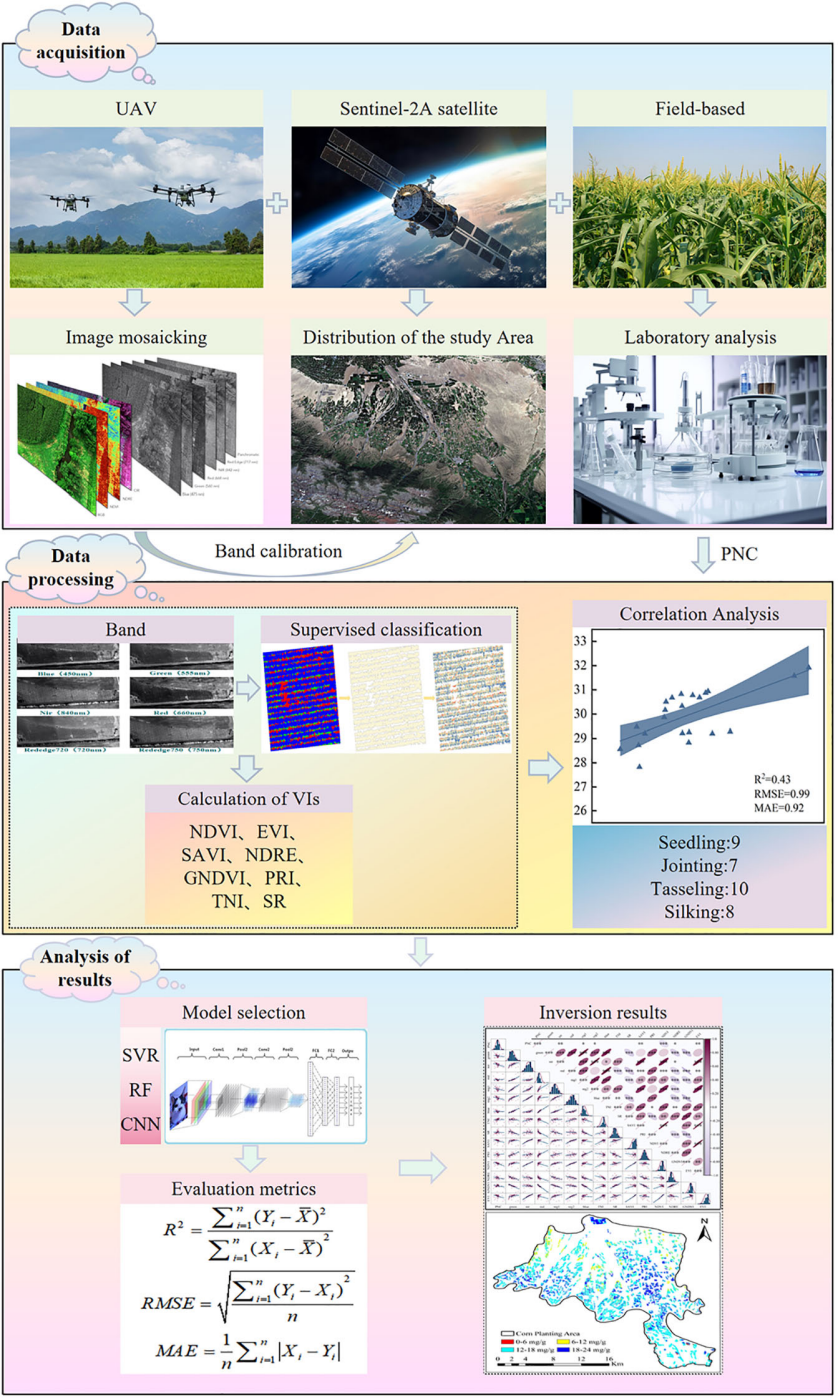
Schematic diagram of the main research workflow.

The overall experimental workflow is illustrated in **Figure 5**, encompassing key steps such as data acquisition, sample collection, image preprocessing, model construction, and accuracy validation, thereby systematically presenting the technical route and implementation process of this study.

## 3 Research methods

### 3.1 Spectral band correction

In multi-source data integration, it is essential to address systematic discrepancies among different sensors by applying band registration and reflectance normalization, thereby ensuring radiometric consistency across multi-temporal and multi-platform datasets (Roy et al., 2014). In this study, UAV-based multispectral data were used to calibrate the reflectance of satellite imagery. The reflectance correction coefficient (C) was calculated using the ratio-mean method, and its expression is shown in Equation 1.


(1) $$C=\frac{\sum_{\text{i}=1}^{n}B_{UAV}/B_{Sentinel-2A}}{n}$$

Here, $B_{UAV}$ and $B_{Sentinel-2A}$ respectively represent the spectral reflectance values at each sampling point obtained from UAV and satellite imagery; n denotes the number of samples (n = 66).

Finally, the correction coefficients were applied to each spectral band of the satellite imagery to achieve consistent reflectance adjustment. By leveraging high-precision UAV data at the local scale, this method effectively compensated for systematic errors in satellite imagery, reduced interference from heterogeneous land surfaces, and significantly improved the consistency of multi-source remote sensing data. It provides a reliable technical foundation for studies requiring the integration of multi-source remote sensing data, such as agricultural monitoring and ecological environment assessment.

### 3.2 Calculation of VIs

VIs are quantitative indicators constructed through the combination and calculation of spectral bands from multi-spectral remote sensing data, based on the strong absorption characteristics of vegetation in the visible red band and the high reflection characteristics in the near-infrared band (Huete et al., 2002). The construction of VIs effectively enhances the spectral signals of vegetation features, while suppressing the interference from environmental factors such as soil background reflection, atmospheric conditions, and canopy structure variations. This spectral feature optimization approach significantly improves the correlation between vegetation physiological parameters and spectral responses, thereby enhancing the accuracy and robustness of remote sensing inversion models (Zarco-Tejad et al., 2013).

Over 40 VIs currently defined have been widely applied in agricultural yield estimation, ecological environment monitoring, and global climate change research (Pettorelli et al., 2005). In this study, by integrating multispectral data and referencing existing literature on PNC inversion, six specific spectral band reflectances and eight commonly used VIs were selected as inversion parameters to construct a maize PNC estimation model. The calculation methods for the selected VIs are provided in **Table 2**.

**Table 2:** Calculation of VIs.

Vegetation index	Name	Formula
NDVI	Normalized Difference Vegetation Index	(NIR-RED)/(NIR+RED)
EVI	Enhanced Vegetation Index	2.5(NIR-RED)/(NIR + 6RED-7.5BLUE+1)
SAVI	Soil-Adjusted Vegetation Index	1.5(NIR-RED)/(NIR+RED+0.5)
NDRE	Normalized Difference Red Edge Index	(NIR-REG2)/(NIR+REG2)
GNDVI	Green Normalized Difference Vegetation Index	(NIR-GREEN)/(NIR+GREEN)
PRI	Photochemical Reflectance Index	(GREEN-RED)/(GREEN+RED)
TNI	Triple Nitrogen Index,	REG1/(GREEN+RED+NIR)
SR	Simple Ratio	NIR/RED

### 3.3 Model development and accuracy evaluation

Pearson correlation analysis was employed to select spectral indices significantly correlated with maize plant nitrogen content. Three machine learning models, including CNN, RF, and SVR, were then developed to achieve high-precision estimation of maize plant nitrogen content. The above models were implemented in the MATLAB R2023a environment.

#### 3.3.1 Pearson correlation analysis

Pearson correlation analysis is a classical parametric statistical method used to quantify the linear relationship between two continuous variables (Schober et al., 2018). Its core lies in the calculation of the Pearson product-moment correlation coefficient (r), which precisely evaluates the strength and direction of the linear association between the variables (Puth et al., 2014). The value of r ranges from -1 to 1, where r = 1 indicates a perfect positive correlation, r = -1 a perfect negative correlation, and r = 0 denotes no linear correlation. In the field of vegetation remote sensing, Pearson correlation analysis is widely used as a classical statistical method to evaluate the linear relationships between multispectral/hyperspectral VIs and key physiological and biochemical parameters (Zhu and Woodcock, 2014). By quantifying the correlation coefficients between variables, this method provides a robust quantitative foundation for feature selection and the development of inversion models for physiological and biochemical parameters.

#### 3.3.2 CNN

The CNN regression model extracts local correlations among one-dimensional features through convolutional layers and integrates nonlinear activation, pooling, and regularization mechanisms. This design reduces the number of parameters while enhancing the capability to model complex nonlinear relationships and perform continuous numerical prediction (LeCun et al., 2015). The model uses one-dimensional numerical features (14 × 1) as input. Its architecture consists of an input layer, two one-dimensional convolutional layers (kernel size of 3×1, containing 16 and 32 feature maps, respectively), batch normalization layers, ReLU activation layers, a max-pooling layer, a dropout layer (dropout rate = 0.1), a fully connected layer, and a regression output layer (Li et al., 2024). Model training was conducted using the stochastic gradient descent with momentum (SGDM) optimizer, with an initial learning rate of 0.01. A piecewise learning rate decay strategy was applied during training, where the learning rate was reduced to 0.1 of its previous value every 800 iterations. The maximum number of training epochs was set to 2000, with a batch size of 32, and the ratio of training to testing samples was 7:3. Through the mechanisms of local connectivity and weight sharing, the model improves feature extraction while reducing parameter complexity. Combined with nonlinear activation, pooling downsampling, and regularization techniques, it effectively enhances the modeling ability of nonlinear relationships among input features (Simonyan and Zisserman, 2014).

#### 3.3.3 RF

The RF regression model is an ensemble learning-based method that achieves accurate prediction of continuous variables by training multiple decision trees and averaging their outputs (Breiman, 2001). Each tree is trained on a randomly sampled subset of the training data, and a random subset of features is selected at each split, thereby enhancing the model’s generalization ability and effectively capturing nonlinear relationships between input features and the target variable (Rodriguez-Galiano et al., 2012). In this study, the RF regression model was implemented in MATLAB R2023a using the TreeBagger function to predict normalized numerical feature vectors (14×1). The model ensemble consisted of 100 decision trees, with a minimum leaf size of 5. Each tree was constructed on a bootstrap sample of the training set, with random feature selection applied at each split to capture complex nonlinear interactions. The training set comprised 44 samples, while the test set included 22 samples. Out-of-bag (OOB) error estimation was employed to evaluate generalization performance, and feature importance was simultaneously calculated to quantify the contribution of each feature to the prediction outcomes.

#### 3.3.4 SVR

SVR is a regression method based on the principles of support vector machines, which predicts continuous values by fitting a smooth function in a high-dimensional feature space (Smola and Schölkopf, 2004). In this study, the SVR model was constructed using the svr_trainer and svr_predict functions for training and prediction. The model employs a Gaussian radial basis function (RBF) kernel to map input features into a high-dimensional space, thereby capturing nonlinear relationships among features. At the same time, the penalty parameter C controls model complexity, while the tolerance error ϵ ensures prediction accuracy and reduces the risk of overfitting. The model was trained using normalized numerical feature vectors (Chang and Lin, 2011). The kernel width parameter γ of the RBF kernel was set to 0.5, the penalty parameter C to 400, and the tolerance error ϵ to 2.5×10⁻⁸. After training, predictions were made on both the training and testing sets, and the predicted results were then denormalized to restore the original units.

#### 3.3.5 Accuracy assessment metrics

In this study, the performance of the maize PNC inversion models was systematically evaluated using three statistical metrics: the R^2^, mean absolute error (MAE), and root mean square error (RMSE). Specifically, R^2^ quantifies the proportion of variance in the observed data that is explained by the model, ranging from 0 to 1, with values closer to 1 indicating better model fit. MAE measures the average absolute deviation between the predicted and observed values, while RMSE emphasizes larger errors through squaring, providing a more sensitive indication of prediction accuracy. Lower MAE and RMSE values indicate higher predictive performance. The calculation formulas for the respective indicators are shown in Equations 2–4.


(2) $$R^{2}=\frac{\sum_{i=1}^{n}{\left(Y_{i}-\bar{X}\right)}^{2}}{\sum_{i=1}^{n}{\left(X_{i}-\bar{X}\right)}^{2}}$$

(3) $$RMSE=\sqrt{\frac{\sum_{i=1}^{n}{\left(Y_{i}-X_{i}\right)}^{2}}{n}}$$

(4) $$MAE=\frac{1}{n}\sum_{i=1}^{n}\left|X_{i}-Y_{i}\right|$$

Here, n represents the total number of samples (n=66/48), $\bar{X}$ denotes the observed mean value of the samples, *X_i_* and *Y_i_* represent the observed value and the predicted value of the i-th sample, respectively.

## 4 Results analysis

### 4.1 Correction of satellite band reflectance

**Table 3** presents the statistical characteristics of surface reflectance values for each Sentinel-2A band after multispectral band correction using UAV data. Overall, the red-edge and near-infrared bands (B5, B6, B8) exhibited high sensitivity to changes in maize canopy structure across different growth stages, with pronounced fluctuations in reflectance, indicating strong potential for vegetation monitoring. In contrast, the visible bands (B2, B3, B4) showed more noticeable variations during the seedling and jointing stages, primarily influenced by vegetation growth conditions and soil background effects.

**Table 3:** Reflectance of satellite bands after calibration.

Date	Band					
	B2	B3	B4	B5	B6	B8
Seedling	0.521417	0.659139	0.712565	0.824374	0.819796	0.803147
Jointing	0.169441	0.324399	0.324399	0.633029	0.839118	0.750172
Tasseling	0.325266	0.567473	0.433107	1.025562	1.277488	1.260719
Silking	0.131029	0.224632	0.170413	0.437153	0.777699	0.779021

**Figure 6:**
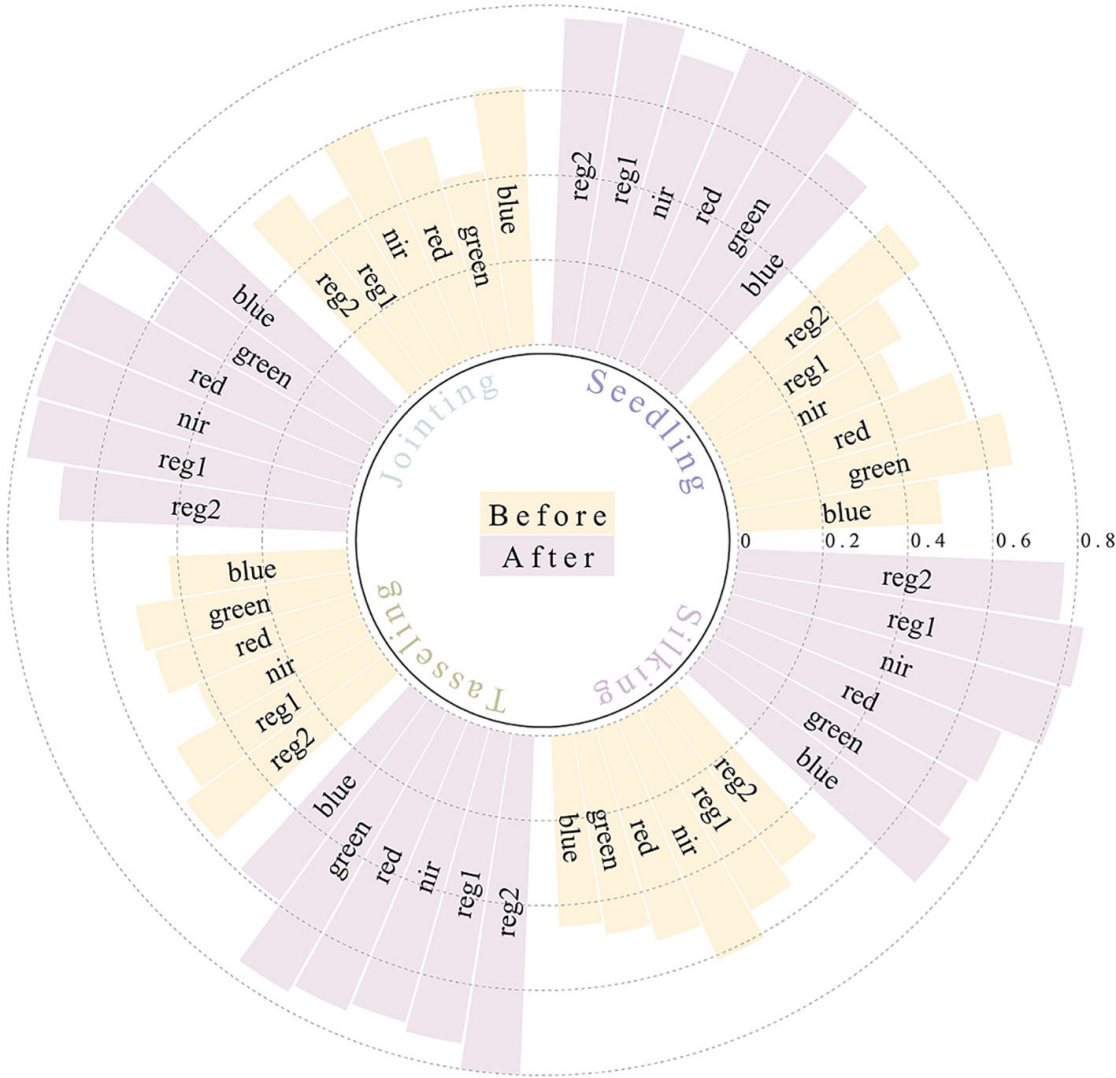
Comparison of R² values for each spectral band of satellite imagery before and after correction.

To evaluate the effectiveness of band correction for Sentinel-2 imagery using UAV data, univariate regression models were constructed for each spectral band before and after correction, and the R² was calculated. As shown in **Figure 6**, the modeling accuracy of the satellite imagery bands was significantly improved after correction using the ratio-averaging method.

### 4.2 Selection of spectral variables

**Figure 7:**
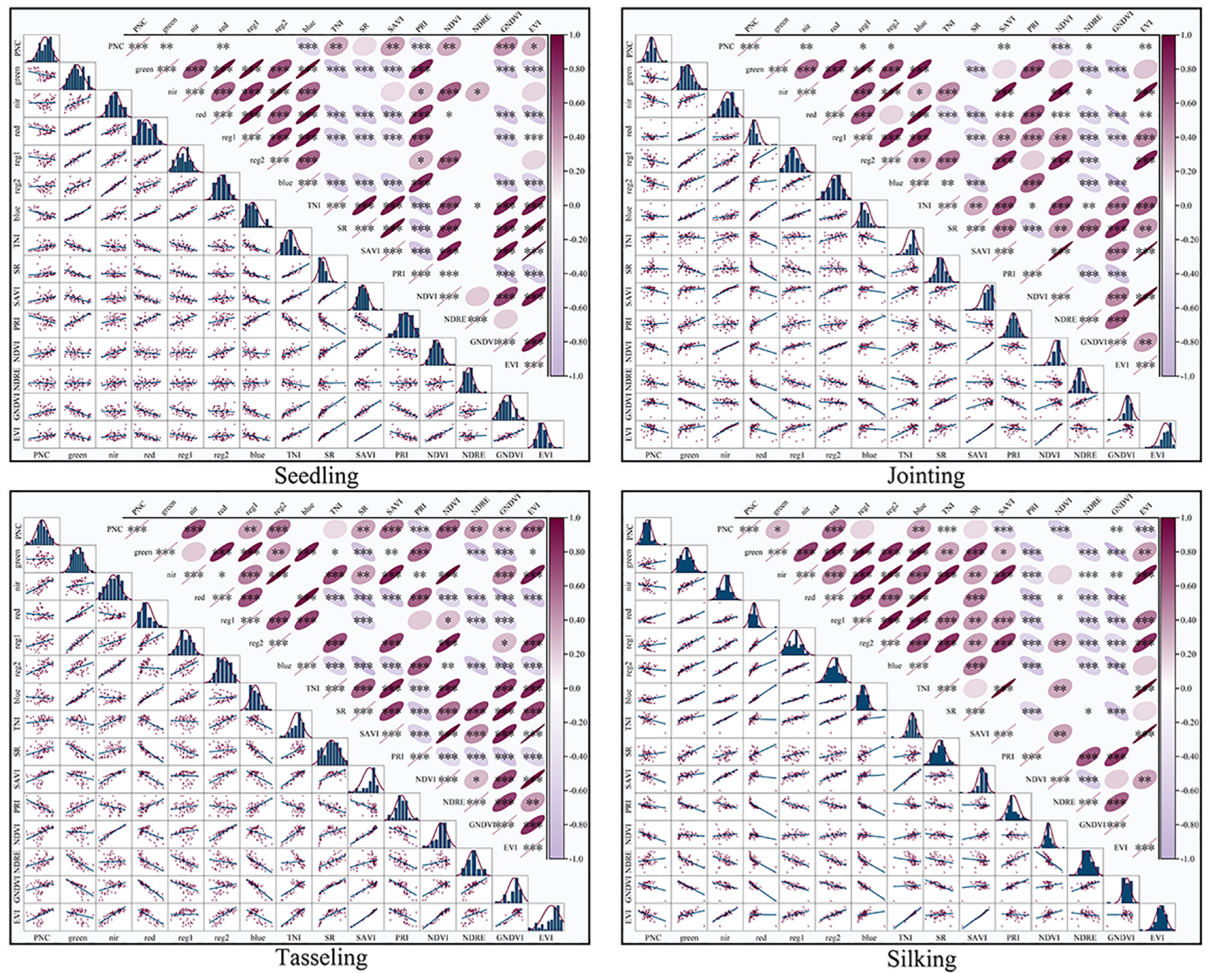
Spectral Variable Selection.

This study systematically selected spectral features significantly correlated with PNC during different maize growth stages based on Pearson correlation analysis, and constructed an inversion model input set with physiological temporal specificity. As shown in **Figure 7**, nitrogen-sensitive variables during the seedling stage are mainly concentrated in the visible light bands (GREEN, RED, BLUE) and wide-band VIs (TNI, SAVI, PRI, NDVI, GNDVI, EVI). Among these, GNDVI exhibits the most significant correlation with PNC, reflecting the dominant role of leaf pigment accumulation in visible light absorption during the seedling stage. After the jointing stage, the sensitive features gradually shift to the near-infrared (NIR) and red-edge bands (REG1, REG2), with a significant increase in the sensitivity of NDVI. This is mainly due to the synergistic effect of rapid canopy expansion and nitrogen metabolism, resulting in dynamic response amplitudes of NIR reflectance and red light absorption reaching peak values during the growth stage. During the tasseling stage, spectral ratio indices (SR) are introduced to strengthen the synergistic response between canopy structure parameters and nitrogen metabolism; during the silk stage, the focus returns to the visible light-dominated mode, with TNI becoming the key variable by quantifying nitrogen transfer efficiency in senescing leaves.

The results demonstrated that the red-edge bands and their derived VIs exhibited significant correlations with maize PNC across different growth stages, with the strongest correlations observed during the jointing and silking stages. This indicates the high applicability of red-edge spectral features in remote sensing-based PNC estimation for maize. The temporal variation in correlation aligns closely with the physiological transition of maize from vegetative to reproductive growth. Further analysis revealed that red-edge parameters showed particularly strong correlations with PNC during the peak vegetative growth phase, confirming their close coupling with chlorophyll content and nitrogen metabolism. This also highlights the intrinsic link between photosynthetic efficiency and nitrogen utilization in maize during vegetative growth. These findings provide a theoretical foundation and technical support for PNC monitoring using red-edge spectral information.

### 4.3 Estimation of maize PNC based on multi-source data

**Figure 8:**
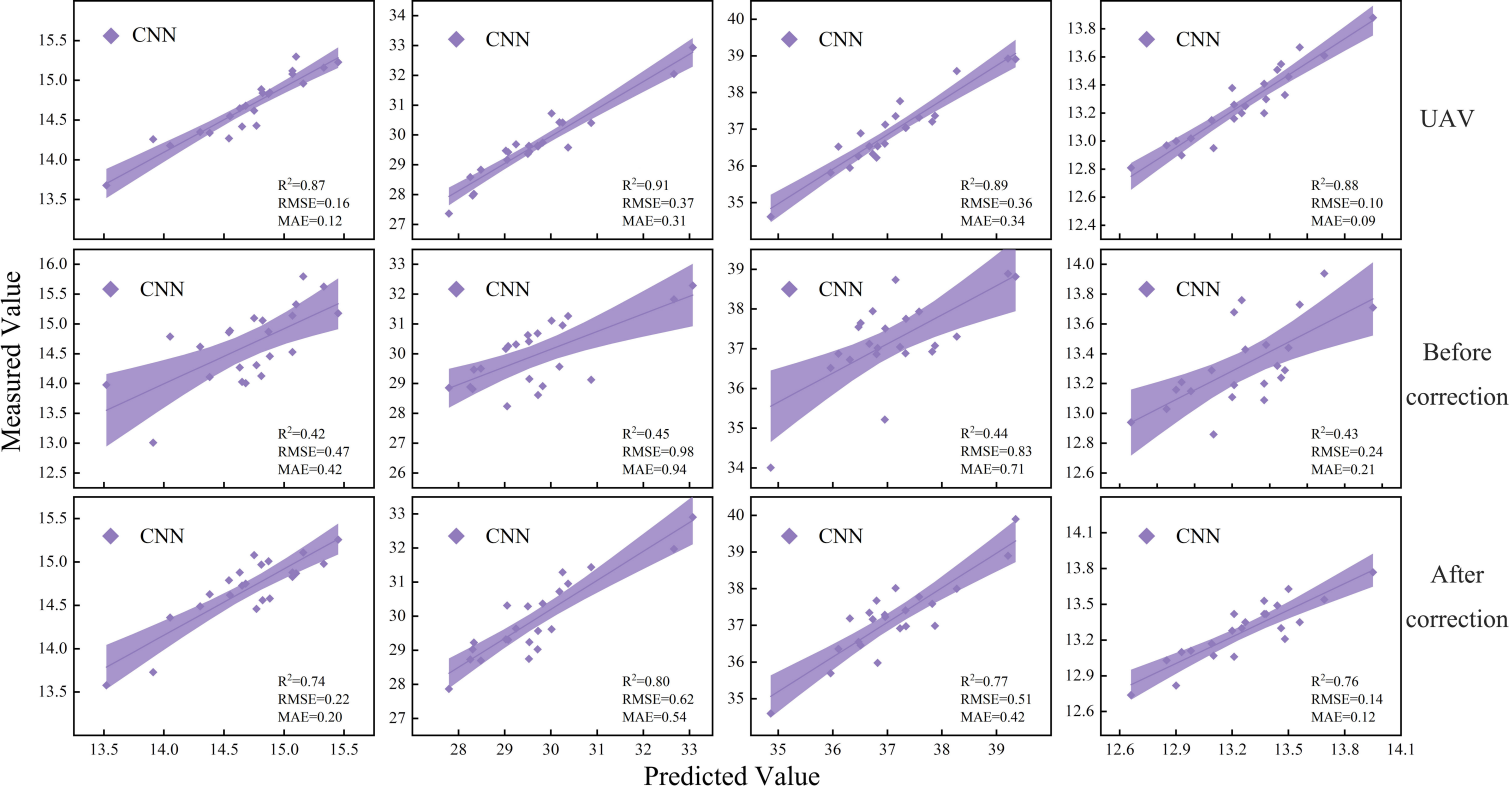
Linear fitting of maize PNC based on the optimal model.

Spectral feature variables significantly correlated with PNC at different maize growth stages were selected as model inputs using Pearson correlation analysis. The shaded areas in the figure represent the 95% confidence intervals, and subfigures (a)-(d) correspond to the four key growth stages. Based on UAV multispectral data, the prediction performance of three machine learning models-SVR, RF, and CNN-was systematically evaluated for PNC inversion during the critical growth stages of maize. As shown in **Figure 8**, the CNN model exhibited the highest prediction accuracy and stability.

Each subfigure presents the comparison of R², RMSE, and MAE values for the CNN model across different validation datasets. The results indicate that model performance varied significantly across different growth stages and input variable conditions depending on the machine learning algorithm used. Among them, the CNN model consistently outperformed both RF and SVR models across all evaluation metrics, demonstrating significantly higher prediction accuracy. The superior performance of the CNN model confirms its advantage in capturing the complex nonlinear relationships between spectral features and physiological parameters. However, its relatively high computational complexity must still be considered in practical applications. These findings provide a scientific basis for evaluating the applicability of different machine learning algorithms in the inversion of crop physiological parameters.

### 4.4 Inversion of maize PNC based on satellite data before and after correction

Compared to the high-precision prediction data obtained from UAVs, the uncalibrated satellite remote sensing data exhibits significant deficiencies in prediction accuracy. Through comparative analysis, the optimal prediction value from the uncalibrated satellite data was about 50.6% lower than that from the UAV data, indicating a clear inadequacy in its predictive capability. Further evaluation of the error metrics revealed that the RMSE of the satellite data reached 1.03 and the MAE was 0.95, both of which were notably higher than the UAV data, reflecting the limitations of satellite data in terms of prediction accuracy. This accuracy gap primarily arises from the inherent limitations of satellite data in spatial resolution, temporal resolution, and data acquisition stability, which hinder its ability to capture subtle surface changes and perform real-time monitoring.

The inversion accuracy of the satellite data was significantly improved through the correction process, as shown in **Table 4**. The R² value increased from the range of 0.35-0.45 before correction to 0.70-0.80 after correction. The average R² value rose significantly from 0.40 to 0.75, representing an 85.7% increase. The maximum RMSE decreased significantly from 1.03 to 0.66, and the minimum MAE reached as low as 0.12, indicating that the band correction process significantly enhanced the predictive accuracy of the satellite data.

**Table 4:** Comparison of model accuracy before and after calibration.

Growth stage		Model	R^2^	RMSE	MAE
Seedling	Before correction	SVR	0.35	0.76	0.71
		RF	0.38	0.58	0.53
		CNN	0.42	0.47	0.42
	After correction	SVR	0.70	0.28	0.23
		RF	0.71	0.24	0.21
		CNN	0.74	0.22	0.20
Jointing	Before correction	SVR	0.41	1.03	0.95
		RF	0.43	0.99	0.92
		CNN	0.45	0.98	0.94
	After correction	SVR	0.72	0.66	0.59
		RF	0.74	0.64	0.49
		CNN	0.80	0.62	0.54
Tasseling	Before correction	SVR	0.37	0.80	0.64
		RF	0.42	0.80	0.73
		CNN	0.44	0.83	0.71
	After correction	SVR	0.71	0.53	0.48
		RF	0.72	0.54	0.47
		CNN	0.77	0.51	0.42
Silking	Before correction	SVR	0.37	0.23	0.20
		RF	0.42	0.23	0.15
		CNN	0.43	0.24	0.21
	After correction	SVR	0.70	0.18	0.15
		RF	0.71	0.16	0.13
		CNN	0.76	0.14	0.12

### 4.5 Setting of auxiliary validation zones and assessment of model applicability

**Figure 9:**
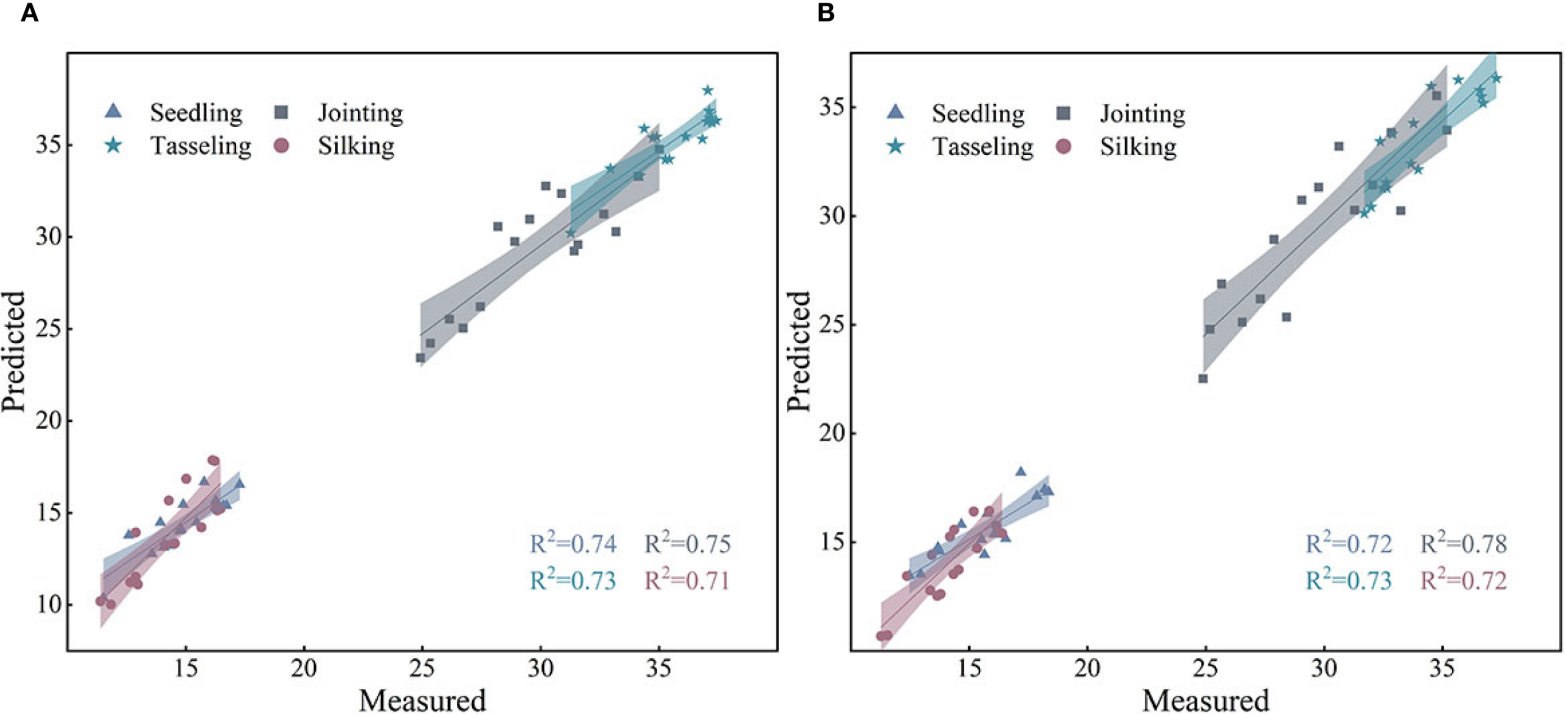
**(A)** Validation accuracy at site B. **(B)** Validation accuracy at site C.

To ensure the scientific validity of the experimental methodology and the reliability of the results, two independent auxiliary validation zones were established in this study. The selection criteria were uniform crop growth, intact canopy, and geographical independence from the main experimental area. A total of 48 samples were collected in the auxiliary validation areas, covering typical spatial heterogeneity of the plots to reflect crop growth differences at the regional scale. By using satellite imagery data calibrated with UAV-based data, PNC inversion validation was conducted in the auxiliary validation zones, as shown in **Figure 9**. This process not only effectively evaluated the applicability and robustness of the constructed model across different spatial extents but also further validated the feasibility and potential for the widespread application of the multi-source remote sensing data fusion method.

To further validate the stability and generalizability of the constructed model, this study performed an inversion accuracy analysis of maize plant nitrogen content (PNC) based on two auxiliary validation sites (Site B and Site C). The figure presents the fitting relationships between the predicted and measured PNC values for each growth stage using the optimal model. At Site B, the prediction results for all four growth stages demonstrated high consistency, with R² values of 0.74, 0.75, 0.73, and 0.71, indicating good predictive performance across different growth stages. At Site C, the overall fitting accuracy improved further, with R² values reaching 0.72, 0.78, 0.73, and 0.72 for each stage. The highest inversion accuracy was observed during the jointing stage, highlighting the model’s exceptional predictive ability for PNC.

Based on the validation results from Sites B and C, it is evident that the use of satellite data calibrated with UAV-based data enables stable inversion of maize PNC across different spatial extents and growth stages, providing reliable technical support for large-scale nitrogen nutrition monitoring in the future.

### 4.6 Inversion of maize PNC in Minle County

**Figure 10:**
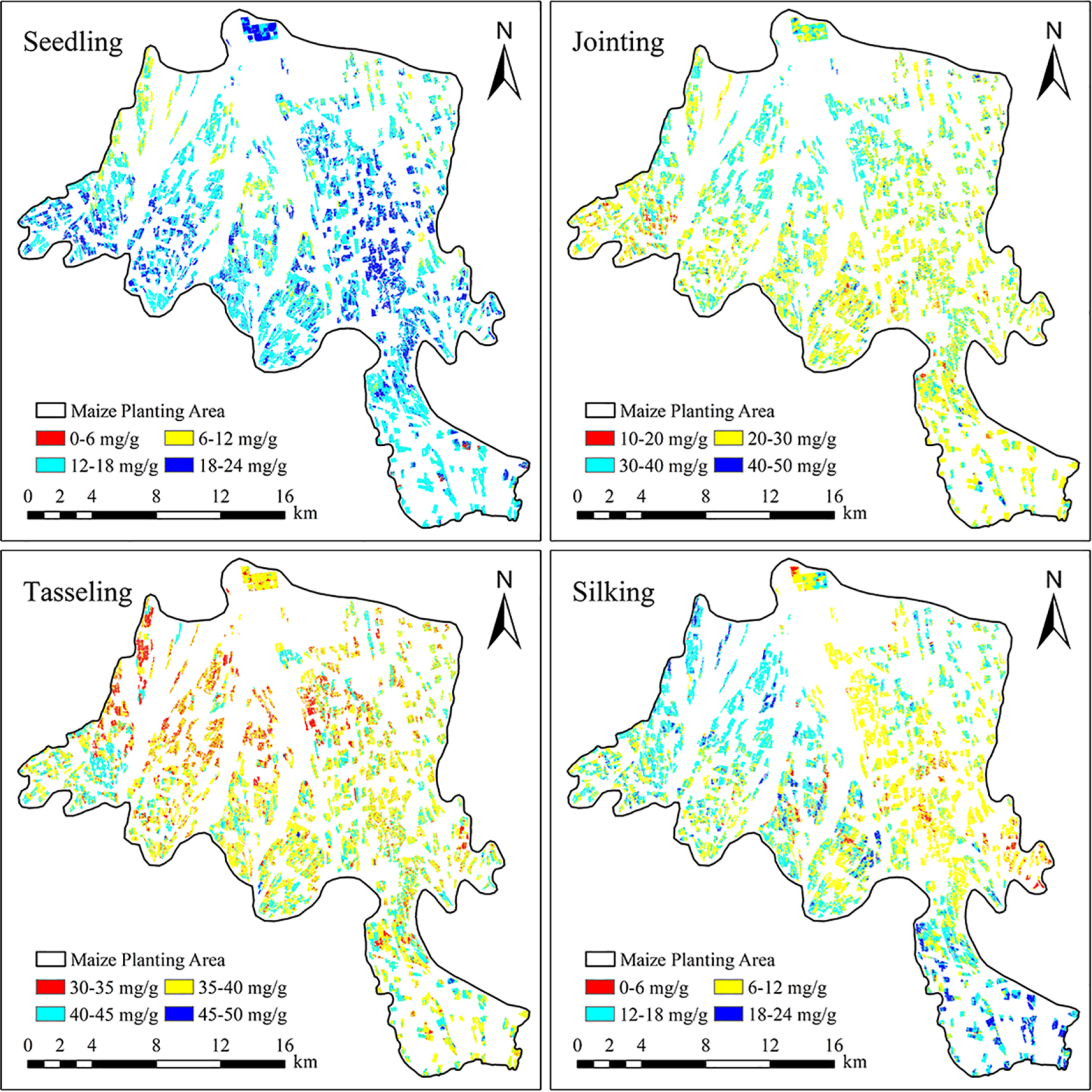
Spatial distribution of maize planting areas in Minle County.

Based on the above analysis, this study further conducted an inversion of maize PNC in the maize cultivation area of Minle County, with the results shown in **Figure 10**. The PNC inversion model, constructed using multi-source remote sensing data and spectral index selection, demonstrated good predictive performance in Minle County, with an overall R² of 0.77, RMSE of 0.54, and MAE of 0.42, indicating that the model can accurately reflect nitrogen content in maize across different growth stages. **Figure 10** illustrates the spatial distribution of maize PNC across four different growth stages in the maize cultivation area of Minle County. The comparative analysis reveals that the figure accurately reflects the spatial variations and changing patterns of maize PNC at each growth stage, aligning closely with the actual field survey results. Overall, regions with higher nitrogen content are concentrated in areas with better soil fertility and irrigation conditions, while regions with lower nitrogen content are predominantly found in plots with less intensive management or insufficient water supply. This spatial distribution pattern is consistent with the actual maize cultivation conditions in Minle County, further validating the reliability and practicality of the inversion model established in this study. Additionally, it provides important insights for future regional nitrogen management and precision fertilization efforts.

## 5 Discussion

### 5.1 The role of spectral index selection in improving model performance

In remote sensing data modeling and inversion studies, feature selection is a crucial step for improving model performance. It effectively removes redundant variables, reduces multicollinearity issues, and enhances the stability and generalizability of the model (Li et al., 2011). This study employed Pearson correlation analysis was used to systematically select spectral indices with the aim of identifying key indicators highly correlated with Maize PNC. By calculating the correlation coefficients between each spectral index and the measured nitrogen content data, spectral features with strong correlations and explanatory power were selected, while indices with high redundancy or weak correlations were excluded. This effectively simplified the model’s input variables and reduced the impact of multicollinearity (Ranjan et al., 2012). The results indicated that using the selected spectral indices as modeling features significantly improved the performance of the PNC inversion model, with the training set R² increasing from 0.78 to 0.83 and the RMSE decreasing from 0.41 to 0.26.

Previous studies have also supported the effectiveness of feature selection in improving model performance. Pearson correlation analysis is widely used in spectral variable selection due to its simplicity in calculation and ease of interpretation. Firouzi et al. (2013) proposed a predictive correlation selection method in high-dimensional data analysis, which is based on Pearson correlation for variable selection. This method significantly reduces prediction errors and enhances model stability. Kaur et al. (2015) systematically evaluated the correlations between over 30 hyperspectral vegetation indices and crop growth parameters, confirming the effectiveness of this method in dimensionality reduction. Ge et al. (2019) further utilized Pearson correlation analysis to select spectral bands and vegetation indices closely related to leaf chemical properties, significantly enhancing the application efficiency of high-throughput spectroscopy in crop monitoring. By selecting features that are highly correlated with the target variable, the model not only captures key variations more accurately but also reduces the risk of overfitting (Bolón-Canedo et al., 2016). These studies collectively indicate that Pearson correlation analysis, as an effective tool for feature variable selection, can significantly optimize the processing workflow of hyperspectral data, providing reliable technical support for crop growth monitoring.

### 5.2 The impact of satellite data band correction on accuracy improvement

When using satellite data to invert Maize PNC, the issue of low accuracy is often encountered. This study proposed a band calibration method by integrating UAV and satellite data, effectively improving the accuracy and reliability of regional-scale nitrogen content inversion. The results indicate that the calibrated satellite data achieved higher accuracy in estimating maize PNC, significantly reducing errors caused by insufficient spatial resolution and spectral response deviations, thereby providing a feasible approach for large-scale agricultural nitrogen monitoring. Traditionally, due to the relatively low spatial resolution of satellite data, which fails to effectively capture subtle changes in crop growth and the spatial heterogeneity of nitrogen content (Blatchford et al., 2020). Additionally, the spectral response of satellite imagery and atmospheric effects may introduce biases in the inversion model when estimating Maize PNC. Although satellite data offers significant advantages for large-scale monitoring, its accuracy is limited by factors such as spatial resolution and spectral response, which restricts its application at the field scale.

To overcome the limitations of UAV data, which has high accuracy but limited coverage, and satellite data, which covers a wide area but has lower accuracy, this study proposes a band correction method that combines both UAV and satellite data, effectively improving the inversion accuracy. By comparing and calibrating the high-precision UAV data with satellite data, the spectral response and spatial resolution of the satellite data were optimized, significantly enhancing the accuracy of the inversion model. Zhang and Zhao (2019) used high-precision UAV imagery as a medium-scale bridge to effectively calibrate the spectral response differences of satellite data, improving the spatial consistency and spectral reliability of remote sensing images in the inversion of coastal saline-alkali soil parameters. Yang et al. (2023) performed dual correction of satellite imagery using UAV data, addressing both spatial and spectral characteristics. This effectively reduced the systematic bias caused by scale differences and enhanced the model’s generalization capability. This study further confirms that integrating multi-platform remote sensing data can overcome the limitations of traditional single-source data, providing a novel technical approach for large-scale agricultural monitoring. By using satellite data calibrated through band correction, it maintains the broad coverage of satellites while achieving accuracy comparable to UAV data. This approach offers solid data support for large-scale agricultural monitoring, precision fertilization, and crop growth monitoring, thus advancing precision agriculture towards larger scales and higher efficiency.

### 5.3 The Impact of different inversion models on the estimation accuracy of maize PNC

The performance of different machine learning models in maize PNC remote sensing inversion in this study shows significant differences. In this study, CNN achieved the highest prediction accuracy within the multi-source remote sensing data framework due to its powerful spatial feature extraction capability (R^2^ = 0.80). It is particularly suitable for handling complex nonlinear relationships in multisource remote sensing data, although it requires high data volumes and computational resources (Lalitha and Latha, 2022). The RF model excels in interpretability and robustness, effectively processing high-dimensional features and assessing variable importance (Zheng et al., 2025). However, its inversion accuracy was slightly lower than that of CNN, with limitations in learning deep spatial features (R^2^ = 0.74). The SVR model is stable in small-sample scenarios, but has limited capacity for processing high-dimensional remote sensing data, making it less suitable for complex feature space relationships (Mountrakis et al., 2011).

The differences in model performance primarily stem from the algorithmic characteristics and the ability to adapt to remote sensing data (Song et al., 2024). The strength of the CNN lies in its capacity to automatically learn multi-level spatial features, giving it a natural advantage in remote sensing image analysis tasks. RF relies on an ensemble decision mechanism to suppress overfitting, ensuring the model’s robustness. In contrast, the SVR model limited by its kernel function mechanism, performs relatively poorly in complex remote sensing inversion tasks. The results of this study indicate that, for farmland nitrogen monitoring applications, models with feature learning capabilities, such as CNN, should be prioritized to fully leverage the information potential of multi-source remote sensing data.This study innovatively combines UAV and satellite remote sensing data, constructing a high-accuracy Maize PNC inversion model through spectral band correction and feature selection. It successfully achieved precise nitrogen monitoring at the regional scale. However, the model still has certain limitations in practical applications. Its performance is highly dependent on the quality of UAV data and can be affected by environmental factors such as cloud cover, canopy shadows, and soil moisture. In addition, the model’s adaptability to crop nitrogen responses at different growth stages remains limited, which may lead to estimation deviations during critical phenological transitions. Future research will focus on integrating multi-source data, including meteorological and soil information, to mitigate the effects of environmental noise and phenological variations, thereby further enhancing the model’s generalizability and robustness in complex agricultural scenarios.

## 6 Conclusion

This study successfully developed a high-accuracy Maize PNC estimation model by synergistically integrating UAV and satellite multi-source remote sensing data. The model was optimized using spectral band correction and feature variable selection techniques, achieving precise monitoring at the regional scale. The study area covers approximately 15,000 hectares of maize cultivation within Minle County, Gansu Province, China, involving multiple large-scale experimental farms. Compared with previous studies, the main innovation of this work lies in proposing a UAV-satellite data synergistic correction framework for crop nitrogen content inversion, which addresses the systematic errors associated with single remote sensing platforms during scale transformation and provides a new technical approach for regional-scale agricultural remote sensing monitoring. Model validation indicates that it performs well for county-level agricultural remote sensing monitoring and can provide a technical reference for similarly sized intensive farming regions. The study demonstrates that:

1. Feature selection plays a key role in enhancing the inversion accuracy of the model. By selecting and optimizing features, data redundancy and noise interference can be significantly reduced, thereby improving the model’s ability to identify key features and its fitting performance.
2. Through a systematic evaluation of three machine learning models, the results indicate that CNN outperforms the other two models in terms of prediction accuracy, with an R² range of 0.74 to 0.80, significantly higher than that of the other models.
3. Band correction significantly improved the PNC inversion accuracy of the Sentinel-2A satellite remote sensing data. The R² of the prediction model increased substantially from 0.35-0.45 before correction to 0.70-0.80 after correction, with an average increase of 88.9%. Due to its high spatial resolution, UAV data can serve as a reference for band correction of satellite imagery, effectively eliminating system errors caused by sensor discrepancies and environmental interferences. This significantly enhances the accuracy and reliability of satellite data, providing more precise remote sensing data sources for earth observation and environmental monitoring.

In summary, this study systematically proposed and validated a collaborative inversion framework for maize PNC by integrating UAV and satellite remote sensing data. Within this framework, high-resolution UAV data were employed to perform band correction on satellite imagery, effectively addressing the limitations of medium- and low-resolution remote sensing data in terms of spatial and spectral accuracy at the field scale, thereby enabling accurate estimation of maize nitrogen content at the regional scale. This technical approach not only significantly enhances the accuracy and reliability of remote sensing inversion but also provides a transferable methodological reference for the synergistic application of multi-platform remote sensing data in agricultural monitoring. Despite the above achievements, this study has certain limitations. The research area is located in a typical irrigated district of the Hexi Corridor, where the climate conditions and cropping patterns are region-specific. Therefore, the general applicability of the results still needs to be validated in other climatic zones and diversified planting environments. In addition, the use of single-year data fails to fully capture interannual variations caused by meteorological fluctuations and field management practices. Future research will focus on incorporating multi-year datasets to enhance the temporal robustness and adaptability of the model.

## Data Availability

The original contributions presented in the study are included in the article/supplementary material. Further inquiries can be directed to the corresponding author.
